# Multilocus Variable Number of Tandem Repeat Analysis Reveals Multiple Introductions in Spain of *Xanthomonas arboricola* pv. *pruni*, the Causal Agent of Bacterial Spot Disease of Stone Fruits and Almond

**DOI:** 10.1371/journal.pone.0163729

**Published:** 2016-09-26

**Authors:** Pablo López-Soriano, Karine Boyer, Sophie Cesbron, María Clara Morente, Javier Peñalver, Ana Palacio-Bielsa, Christian Vernière, María M. López, Olivier Pruvost

**Affiliations:** 1 Instituto Valenciano de Investigaciones Agrarias, Moncada, Valencia, Spain; 2 UMR Peuplement Végétaux et Bioagresseurs en Milieu Tropical, Centre de Coopération Internationale en Recherche Agronomique pour le Développement, Saint-Pierre, La Réunion, France; 3 INRA, UMR1345 IRHS Institut de Recherche en Horticulture et Semences, Beaucouzé, France; 4 Centro de Investigación y Tecnología Agroalimentaria de Aragón. Instituto Agroalimentario de Aragón, IA2 (CITA-Universidad de Zaragoza), Zaragoza, Spain; 5 UMR Biologie et Génétique des Interactions Plante-Parasite, Centre de Coopération Internationale en Recherche Agronomique pour le Développement, Montpellier, France; Virginia Tech, UNITED STATES

## Abstract

*Xanthomonas arboricola* pv. *pruni* is the causal agent of the bacterial spot disease of stone fruits, almond and some ornamental *Prunus* species. In Spain it was first detected in 2002 and since then, several outbreaks have occurred in different regions affecting mainly Japanese plum, peach and almond, both in commercial orchards and nurseries. As the origin of the introduction(s) was unknown, we have assessed the genetic diversity of 239 *X*. *arboricola* pv. *pruni* strains collected from 11 Spanish provinces from 2002 to 2013 and 25 reference strains from international collections. We have developed an optimized multilocus variable number of tandem repeat analysis (MLVA) scheme targeting 18 microsatellites and five minisatellites. A high discriminatory power was achieved since almost 50% of the Spanish strains were distinguishable, confirming the usefulness of this genotyping technique at small spatio-temporal scales. Spanish strains grouped in 18 genetic clusters (conservatively delineated so that each cluster contained haplotype networks linked by up to quadruple-locus variations). Furthermore, pairwise comparisons among populations from different provinces showed a strong genetic differentiation. Our results suggest multiple introductions of this pathogen in Spain and redistribution through contaminated nursery propagative plant material.

## Introduction

Geographical expansions of pathogens into new areas are one of the most important factors involved in plant disease emergence [1]. Such introductions have become more frequent over the last decades due to major increases in plant trade and transport [2], which has favored the exchange of contaminated plant material. The application of efficient molecular typing tools to the study of bacterial populations becomes essential for a better understanding of their genetic diversity and may allow the reconstruction of the invasion routes in order to develop preventive control strategies.

*Xantomonas arboricola* pv. *pruni* [3] (synonym, *Xanthomonas campestris* pv. *pruni* Smith) is the causal agent of bacterial spot disease of stone fruits, almond and some ornamental species of *Prunus*. It is considered one of the most important bacterial pathogens affecting plants of the genus *Prunus* due to its high economic impact since most of the commercial cultivars of peach, Japanese plum, apricot and almond are susceptible [4, 5]. Crop losses may exceed 10,000 € per ha in plum orchards in favorable years to the development of the disease [4] and, in Spain, decrease production in susceptible almond cultivars may reach 47% [6]. For these and other reasons, this bacterium is listed as a quarantine organism in the European Union (EU) phytosanitary legislation (EU Council Directive 2000/29/EC) and in the European and Mediterranean Plant Protection Organization (EPPO) list (EPPO A2 list).

The disease was first described in North America in 1903 on Japanese plum [7] and thereafter *X*. *arboricola* pv. *pruni* has been reported in the main stone fruit producing areas from the five continents [8]. In Europe this pathogen is present in Italy, where it is considered endemic [9] and it has emerged in Belgium, France, Germany, the Netherlands, Switzerland, Spain and some Eastern Europe countries [10] (EPPO-PQR, 2015). In the Americas, it is endemic in many areas of Argentina, Brazil, United States and Uruguay. Symptoms are observed on leaves and fruits, and may cause dieback [8, 11]. The severity of disease outbreaks was found strongly related to weather conditions, cultivar susceptibility and agricultural practices. Bacteria survive during winter in cankers, leaf scars and buds in the absence of symptoms [9, 12, 13]. Shepard and Zehr [14] also showed the epiphytic persistence of this pathogen in different plant organs in population ranging from 10^2^ to 10^6^ CFU per gram of fresh weight in asymptomatic plant material. As there is no effective chemical control available, and to be part of a sustainable management, integrated control strategies are advised. In this context, it is essential to control the sanitary status of plant material in nurseries in order to propagate it free from the disease [4]. Currently, in the EU legislation, a visual inspection in nurseries for symptoms is the only requirement to certify plants free of *X*. *arboricola* pv. *pruni*, but latent infections and/or epiphytic populations in asymptomatic propagative material have been found [13, 15, 16].

Despite its economic importance, little is known about the population biology and epidemiology of this quarantine bacterium. Boudon et al. [17] used fluorescent amplified fragment length polymorphism (FAFLP) analysis and multilocus sequence analysis (MLSA) to study the diversity of 64 *X*. *arboricola* pv. *pruni* strains isolated from different countries worldwide. No polymorphism was observed by MLSA based on four gene portions indicating that *X*. *arboricola* pv. *pruni* is a monomorphic bacteria [18]. Subsequent studies based on seven gene portions highlighted minor polymorphism but further confirmed its monomorphic nature [19]. Similarly, FAFLP globally yielded little polymorphism, slightly larger in America than in Europe and consistent with the history of the reported outbreaks. Similar results were obtained by Barionovi and Scortichini [20] when screening the variability of 47 *X*. *arboricola* pv. *pruni* strains collected from Italy, Spain and Australia using an integron gene cassette array and BOX-PCR. Kawaguchi [21] also observed low genetic diversity in *X*. *arboricola* pv. *pruni* Japanese strains using inter-simple sequence repeat PCR and repetitive sequence-based (rep)-PCR. Thus, all these methods lack resolution for deeper genetic diversity analyses at intrapathovar level, especially at small spatio-temporal (e.g. outbreak investigation) scales.

Multilocus variable number of tandem repeat analysis (MLVA) is a reliable genotyping method that proved efficient for assessing the genetic diversity of monomorphic bacterial pathogens [22] and it is based on the variation detected in the size of short repetitive DNA sequences, tandem repeats (TR), which are highly variable regions in bacterial genomes [23]. Variation in TR number is produced mainly by slipped strand mispairing during DNA replication [24]. Most TRs evolve following the stepwise mutation model where new alleles are created by the addition or deletion of a single repeat unit per mutation event [25], or a generalized two-phase model where multiple-repeat mutation frequencies follow a geometric distribution [26]. The first MLVA scheme targeting a bacterial plant pathogen was performed for *Xylella fastidiosa* [27]. In *Xanthomonas* species, MLVA schemes have been developed first for *X*. *citri* pv. *citri* [28, 29] and later on for *X*. *oryzae* [30, 31] and *X*. *axonopodis* pv. *manihotis* [32]. A comprehensive MLVA scheme targeting 26 TR loci was developed to discriminate 61 *X*. *arboricola* strains of different pathovars (*celebensis*, *corylina*, *fragariae*, *juglandis*, *poinsetticola*, *populi* and *pruni*) [33]. Essakhi et al. [34] used MLVA for assessing the genetic relatedness between *X*. *arboricola* pv. *juglandis* (a walnut pathogen) and non-pathogenic *X*. *arboricola* strains also isolated from walnut. Six TR loci were used to subtype 25 *X*. *arboricola* pv. *pruni* isolates from cherry laurel (*P*. *laurocerasus*) in the Netherlands [35].

Here, we study the genetic relatedness among strains causing bacterial spot disease in Spain, where *X*. *arboricola* pv. *pruni* was first detected in 2002 on Japanese plum, and in subsequent years several outbreaks have occurred in the main stone fruit producing regions [5, 8]. Our objective was to assess the genetic diversity in a collection of 239 Spanish strains of *X*. *arboricola* pv. *pruni* as well as in 25 reference strains. To achieve this goal, we used a MLVA scheme including two sets of molecular markers combining microsatellite and minisatellite loci. This is the first extensive study targeting the intra-pathovar genetic diversity of a large collection of *X*. *arboricola* pv. *pruni* strains in a single country as an attempt to provide clues for better understanding introduction and dispersal pathways.

## Materials and Methods

### Bacterial strains and DNA extraction

**Fig 1:**
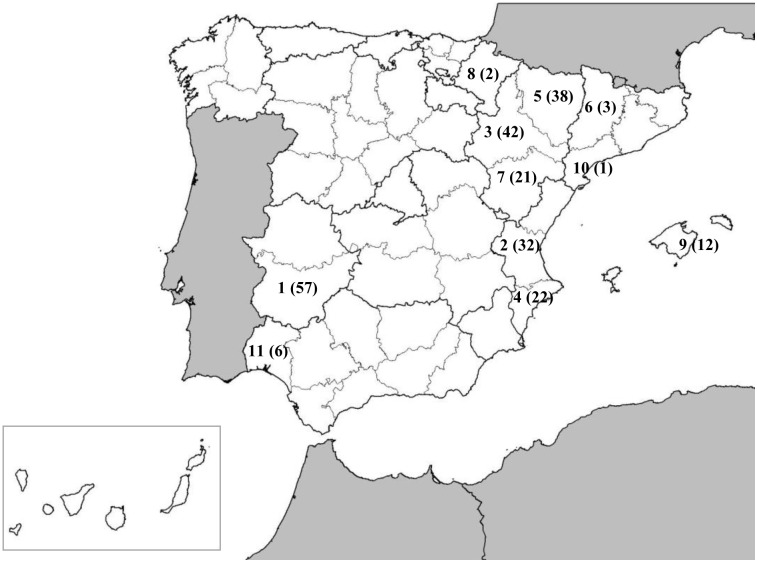
Chronological detection of outbreaks of *Xanthomonas arboricola* pv. *pruni* in Spain. 1, Badajoz, 2002; 2, Valencia, 2003; 3, Zaragoza, 2004; 4, Alicante, 2006; 5, Huesca, 2008; 6, Lleida, 2008; 7, Teruel, 2009; 8, Navarra, 2009; 9, Mallorca, 2010; 10, Tarragona, 2011; 11, Huelva, 2012. In brackets, number of isolates per province.

A total of 264 strains of *X*. *arboricola* pv. *pruni* were used in this study, 239 isolated from 11 Spanish provinces (Alicante, Badajoz, Huelva, Huesca, Lleida, Mallorca, Navarra, Tarragona, Teruel, Valencia and Zaragoza) as shown in Fig 1 and S1 Table. Spanish strains were isolated from commercial orchards, nurseries and experimental plots since the first detection of this pathogen in Spain in 2002 to 2013. The strains used in this study were obtained from samples received as Laboratory of Reference of the Spanish Ministry of Agriculture (IVIA). The Ministry has been consulted about publishing the results and as official laboratory we do not require specific permission for handling these strains.

Strains were routinely cultured on YPGA medium [36] (yeast extract 5 g l^-1^ [Difco], bacteriological peptone 5 g l^-1^ [Difco], glucose 10 g l^-1^ and agar 20 g l^-1^ [pH 7.1]) at 25°C for 72 h. DNA from bacterial pure cultures was obtained using a simple isopropanol-based extraction method, as previously described [37]. DNA concentration and purity were determined by using a ND1000 spectrophotometer (Thermo Fisher, Alcobendas, Spain) and the preparations were stored at -20°C. Most of the strains were isolated from Japanese plum (*Prunus salicina*) (n = 103), almond (*P*. *dulcis*) (n = 77), peach (*P*. *persica*) (n = 39), a few from apricot (*P*. *armeniaca*), nectarine (*P*. *persica* var. *nectarine*), flat peach (*P*. *persica* var. *platycarpa*), and from the rootstocks Cadaman^®^ (*P*. *persica* x *P*. *davidiana*), Barrier (*P*. *davidiana* x *P*. *persica*), Garnem (*P*. *dulcis* x *P*. *persica*) and Monegro (*P*. *dulcis* x *P*. *persica*) (S1 Table). Spanish strains were also compared to 25 representative strains from Argentina, Australia, Brazil, Canada, France, Italy, New Zealand and United States conserved in international collections (S2 Table). A panel test comprising 16 strains representative of the geographical and genetic diversity of the pathogen was used for preliminary primers screening (S1 and S2 Tables).

### Genomic DNA isolation, sequencing and annotation

Genomic DNA from *X*. *arboricola* pv. *pruni* pathotype strain CFBP 3894 was isolated and purified using the Qiagen genome DNA isolation kit (Qiagen, Hilden, Germany) according to the manufacturer’s instructions. The Genomic DNA quality and quantity were assessed on an agarose gel and using a NanoDrop ND-1000 spectrophotometer (the NanoDrop Technologies, Wilmington, DE). Libraries with an average insert size of 350 bp were sequenced using the Illumina HiSeq 2000 platform (Genoscreen, France). Paired-end reads were assembled in contigs using SOAP*denovo* 1.05 [38] and Velvet 1.2.02 [39]. The assemblies had a total length of 5.06 MB generating 77 contigs. The G+C content of the sequence was of 65.42%. Annotation was performed using EuGene-PP using similarities with known protein sequences [40]. Annotation of the genome sequences revealed 4337 putative protein-coding sequences, 55 tRNA, and three rRNA.

### TR selection

In a first step 26 TR loci previously selected by Cesbron et al. [33] were tested with a test panel of 16 Spanish and reference strains from different geographical origins. TR primers were assessed by PCR as follows: 5 to 10 ng of DNA was used as template in a 15 μl reaction mix containing 0.3 μM of each primer, 1X Terra Buffer (Ozyme, Saint Quentin en Yvelines, France), 0.5X Q-solution and 0.375 U Terra Polymerase Mix (Ozyme). All reactions were performed in a Veriti Thermal Cycler (Applied Biosystems, Villebon sur Yvette, France) using the following PCR conditions: initial denaturation at 98°C for 2 min, followed by 35 cycles consisting of denaturation at 98°C for 10 s, annealing at temperatures ranging from 58 to 68°C for 15 s, and extension at 68°C for 1 min, with a final extension step at 68°C for 30 min. PCR products were visualized under UV light in agarose gels (2%) stained with ethidium bromide.

In a second step, the draft genome sequence of the *X*. *arboricola* pv. *pruni* pathotype strain CFBP 3894 was screened for minisatellites using the Tandem Repeat Finder (TRF) (http://minisatellites.u-psud.fr/; http://tandem.bu.edu/trf/tr.html) [41, 42]. Parameters selected were total length in a range of 50–1000 bp and tandem repeats length ≥ 10 bp. Other parameters were set as default. Twenty-three single TR loci were retained from the genome of the CFBP 3894. Primer pairs targeting single locus alleles were designed in the TR flanking regions using Primer3, included in Geneious 7 software. Primers were tested with the test panel strains by PCR with the same conditions described above. Amplicons were visualized as mentioned above.

### MLVA scheme

A total of 23 primer pairs targeting single locus alleles were used in a multiplex PCR format. Primers were grouped in multiplex pools according to their annealing temperature. Each primer in the PCR mix was 5’- labeled with one of the following fluorescent dyes: 6-FAM, NED, PET and VIC (Applied Biosystems) (Table 1). Each PCR reaction contained 5 to 10 ng of genomic DNA as template in 15 μl mix containing 0.3 to 1.2 μM of each primer, 1X Terra Buffer (Ozyme), 0.5X Q-solution and 0.375 U Terra polymerase (Ozyme). PCR amplifications were performed in a Veriti Thermal Cycler (Applied Biosystems) using the following conditions: 2 min at 98°C for polymerase activation, followed by 25 cycles at 98°C for 10 s, annealing temperatures ranging from 64 to 68°C for 15 s (Table 1), and 68°C for 1 min (except in one pool where elongation time was 2 min 30 s), and a final extension step at 68°C for 30 min. 1 μl of diluted amplicons was mixed with 0.1 μl of GeneScan-500 LIZ or 0.5 μl of GeneScan-1200 LIZ internal size standard (Applied Biosystems) and 10.9 μl or 10.5 μl of Hi-Di formamide (Sigma-Aldrich) (for GeneScan-500 LIZ and GeneScan-1200 LIZ, respectively). Capillary electrophoresis was performed in an ABI PRISM 3130xl Genetic Analyzer and results were analyzed with GeneMapper 4.0 (Applied Biosystems).

**Table 1:** TR markers tested on Spanish strains of *X*. *arboricola* pv. *pruni* (n = 239), primers, PCR conditions, number of alleles and Nei’s genetic diversity (H_T_).

Name	TR length (bp)	Primers	Annealing T (°C)	Primer concentration (μM)	PCR pool	Range of repeat numbers	Number of alleles (H_T_)^a^
**TR51I**	7	5’ FAM-CATGGCAGTGCAGGTGGATC 3’ 5’ CTGCAACTCCCGATTCCCGA 3’	68	0.3	1	4–6	3 (0.154)
**TR37I**	7	5’ VIC-ATGGAGGATGCGGTTGCGGCT 3’ 5’ CCAACAGAACCCCGCACCCA 3’	68	0.3	1	4–6	3 (0.384)
**TR37II**	6	5’PET-CGTCATGGACGCCCTGGTCAG 3’ 5’ CATTGGCATCGGCACGGCTACT 3’	68	0.3	1	9–11	3 (0.017)
**TR05-06**	7	5’ NED-GTCGACGGGTTCGCGGAAGGT 3’ 5’ GTGCAGCACCAGCCAAAGGCA 3’	68	0.3	1	15–21	7 (0.795)
**TR40I**	7	5’ FAM-TATCAGGCAGCGCACCAGCT 3’ 5’ TGGAATGTGGAGGCTGTTCG 3’	68	0.3	2	7–8	2 (0.168)
**TR36I**	7	5’ VIC-GCAGGAGAAGGAAAGCGCCAG 3’ 5’ CGATCGCATCTGTGTGGGTTAG 3’	68	0.3	2	5–6	2 (0.132)
**TR03I**	7	5’ PET-GACATTCGCCGGGAGTGCAG 3’ 5’ GGTTGCTTGGTCGTTGATCG 3’	68	0.3	2	7–8	2 (0.008)
**TR68I**	7	5’ NED-CTTGCGGTACTGGCTGTTCA 3’ 5’ AAATCATCGGCGCCTGAAAC 3’	68	0.3	2	9–15	7 (0.632)
**TR30II**	7	5’ FAM-TTCTGCCGTCTTTCAGGGCTGG 3’ 5’ CATCAGTGCGAGGCCACGAAC 3’	66	0.3	3	7–11	4 (0.371)
**TR28II**	9	5’ VIC-GCGGCATGTTCCGACTGCACC 3‘ 5’ GGGTGGATGAGGGTCTGCATG 3’	66	0.6	3	3–4	2 (0.008)
**TR38II**	6	5’ PET-TCTCGGTATCGATGTGGGTGC 3’ 5’ CCCGTAGCTGTATCAGTGCCT 3’	66	0.9	3	6–12	7 (0.691)
**TR58II**	7	5’ NED-GGAAGAGTACCCGGCAATTCT 3’ 5’ TCTGATCGGTGCTGAGCGTCT 3’	66	0.6	3	9–17	8 (0.667)
**TR33I**	7	5’ FAM-CGAGTGGATGTTATGGCGTGG 3’ 5’ CTCGCAAAACCCTTGCCATC 3’	66	0.3	4	5–8	4 (0.207)
**TR58I**	7	5’ VIC-ATCTGTTGCTGGCCGAGAGC 3’ 5’ ACCAACACCGAGCTTGCCTC 3’	66	0.3	4	5–9	5 (0.355)
**TR79I**	7	5’ NED-GCTGATCCTTCGTGGGCTTG 3’ 5’ GGTGTGAATTCGTCGGTGAC 3’	66	0.3	4	6–10	5 (0.393)
**TR50I**	7	5’ VIC-GTTGCGAGATCGGGCGCTTC 3’ 5’ CGTGCATCAGACGCTTGCGT 3’	68	0.3	5	5–9	5 (0.727)
**TR66I**	8	5’ PET-TGCAGTTGTGGTCTTCGGCA 3’ 5’ CACGCATCAAGTTCGACATGGTGC 3’	68	0.3	5	9–12	4 (0.719)
**TR15I**	7	5’ NED-GCCATGTCGCCGGGAAACGA 3’ 5’ TCGAGCGGTTCCTGCGGTTGT 3’	68	0.3	5	6–7	2 (0.285)
**TR67II**	7	5’ VIC-AGATACAAGGCGAACGCGAT 3’ 5’ CAGGACAGGAACGGCAACC 3’	64	0.3	6	10–20	11 (0.779)
**Xap4790**	112	5’ CGCGTATTGCAGGAATCCAC 3’ 5’ FAM-CGATTGGAGATCCGGACCAG3’	68	0.3	A	2–4	2 (0.041)
Xap4422^b^	116	5’ GGTCTGATCCGCTTCTCACC 5’-NED-ATCCGCGCCAACTACAAGAA	68	0.3	A		
**Xap0897**	15	5’ ATTACTTCTTCCCCTGCGGC 3’ 5’ FAM-TCTACAACACCAAGACCGGC 3’	64	1.2	B	1–3	2 (0.008)
**Xap2280**	20	5’ AGAGCCTACACGGACGTACT 3’ 5’ VIC-TGAACGGGATGGTGCAAGTT 3’	64	0.3	B	7–13	6 (0.508)
**TR10II**	12	5’ TGGTTGCGCCCTTGCCTTCTC 3’ 5’ PET-TCGCCGGCATCAACATGGCCG 3’	64	0.9	B	5–7	3 (0.292)

^a^ Nei’s genetic diversity calculated using ARLEQUIN version 3.01.

^b^ This marker was monomorphic within Spanish strains and was only used to compare them to the worldwide collection.

### Data scoring and analysis

Fragment sizes obtained for each TR locus using GeneMapper 4.0 (Applied Biosystems) were transformed to tandem repeat numbers and used as input data. According to Pourcel and Vergnaud [43] when a TR array was truncated, the TR number was rounded up to the higher integer.

Strains sharing the same MLVA profile were grouped in haplotypes. Phylogenetic relationships between strains were studied generating a minimum spanning tree (MST) with PHYLOViZ v1.0 [44]. Clonal complexes (CC) were defined as networks of single locus variants (SLVs, i.e. groups of strains differing at a single TR locus), and were obtained and analyzed with eBURST v3 [45]. The founder was defined as the haplotype with the largest number of SLVs.

The population structure of the collection of the Spanish strains was assessed using PHILOViZ v1.0. A categorical MST was generated using the algorithm recommended for MLVA data combining global optimal eBURST (goeBURST) and Euclidean distances. Genetic clusters (GCs) were delineated so that each cluster contains a network of haplotypes linked by up to quadruple-locus variations.

### Genetic diversity of Spanish strains

Only Spanish strains were used to calculate genetic diversity indices and to perform analysis of population structure at the province level, although only those provinces where more than 12 strains were isolated were selected (i.e. 224 strains from seven provinces) which represent 94% of total strain collection studied. Nei’s unbiased estimates of genetic diversity (H_E_) were calculated using ARLEQUIN version 3.01 [46]. Allelic richness (A), the mean number of alleles per locus per population, was calculated using the rarefaction procedure with HP-RARE version 1.0 [47]. Genetic differentiation among provinces was evaluated with Wright’s fixation index (F_ST_) and Slatkin’s F_ST_ analogue (R_ST_), which were calculated by computing distance matrices for haplotypic data. Significance was tested with 1000 permutations using ARLEQUIN version 3.1. [46].

Metric multidimensional scaling (MDS), based on a Manhattan distance matrix, was used to represent distances among the Spanish and the worldwide strains. MDS transforms a distance matrix (which cannot be analyzed by eigen-decomposition) into a cross-product matrix, then solving the eigen-vector problem to find the coordinates of individuals so that distortions to the distance matrix are minimized. As in principal component analysis, individuals are projected into *n* dimensions [48]. MDS was performed using the cmd-scale function in the R software.

## Results

### Selection of TR markers

The panel test of 16 *X*. *arboricola* pv. *pruni* strains was first tested by the 26 TR loci previously selected by Cesbron et al. [33]. Primers were redesigned when original primers were found to hybridize within the TR array (e.g. TR18II and TR67II) or when mispriming was observed (TR21II). Four TR loci were not further considered (TR19I, TR54I, TR39II and TR18II). In the context of xanthomonads, which often contain compound microsatellites in their genome (i.e. loci for which TRs are composed of two or more sequence types), we considered TRs to be different when they had less than 70% sequence identity. TR19I was found to be a combination of two microsatellites sharing only 58% identity. TR54I showed a combination of two microsatellites (showing only 29% identity) and one minisatellite. TR39II and TR18II showed very different profiles in closely related strains (i.e single- or double-locus variants), in some cases differences were up to 12–14 repeats in the same locus between strains isolated in the same orchard and the same year. Sanger sequencing confirmed that the observed size polymorphism concerned the TRs and not the flanking regions. These differences may be attributed to recombination events and this suggested that these two loci might not follow a stepwise or two-phase mutation model. Therefore, they were not further considered in the analysis.

Moreover, from the genome sequence of strain CFBP 3894, markers TR05II and TR06II corresponded to a same and single tandem repeat locus. Furthermore, TR05II forward primer and TR06II reverse primer were found to prime within the repeated sequence. Therefore, we used the forward primer of TR06II and the reverse primer of TR05II (new marker named TR05-06), which did not prime in the tandem repeat array (S1 Fig).

The panel test of strains was also used to test primer pairs targeting 23 minisatellites identified with TRF screening the draft genome sequence of strain CFBP 3894. Four of them were polymorphic (Xap4790, Xap0897, Xap4422 and Xap2280) and were included in the MLVA scheme, whereas the other markers were monomorphic. Xap4422 was monomorphic among Spanish strains and was only retained to compare Spanish strains to the worldwide collection of strains.

Consequently we eventually retained 23 TR loci (18 microsatellites (TR < 9bp) and 5 minisatellites (TR ≥ 9 bp) [43] for assessing the diversity of the Spanish strains collection and 24 loci for the worldwide collection.

### MLVA genotyping of the Spanish strains

The set of 239 strains of *X*. *arboricola* pv. *pruni* isolated in Spain was successfully genotyped, and all of them yielded reproducible amplicons for all 23 TR loci. Nei’s genetic diversity at each locus ranged from 0.008 to 0.795 (Table 1). Three TR loci (TR03I, TR28II and Xap0897) showed very low levels of polymorphism and differentiated a single isolate in the whole collection. TR37II and Xap4790 were also weakly polymorphic and only differentiated two and four isolates, respectively. Allele number per locus ranged from 2 to 11 (Table 1).

**Fig 2:**
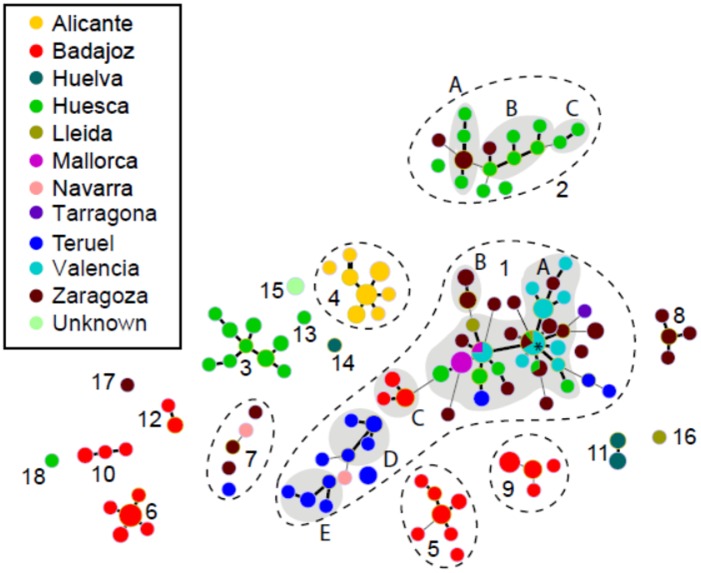
Categorical minimum spanning tree from MLVA data (239 strains and 119 haplotypes) representing the genetic diversity of the Spanish strains of *X*. *arboricola* pv. *pruni* in relation with its province of origin. Dot diameter represents the number of strains per haplotype. Numbers represent the 18 genetic clusters identified. Haplotypes in the same genetic cluster are up to quadruple locus variants. Thick links indicate single locus variants and thin links indicates double locus variants. Shaded areas show different clonal complexes in genetic clusters 1 and 2 and are identified with letters (A-E). Genetic clusters not enclosed in dashed lines are formed by a unique clonal complex or singleton. *Indicates the primary founder haplotype.

Out of 239 Spanish strains, 119 MLVA haplotypes were identified. The genetic diversity of the whole collection was H_T_ = 0.363. Genetic diversity indices for each province are shown in Table 2. In order to assess the population structure of the Spanish strains, a categorical minimum spanning tree was built. Eighteen GCs were obtained grouping haplotypes differing by up to four TR loci (Fig 2).

**Table 2:** Genetic diversity estimated from MLVA data of *X*. *arboricola* pv. *pruni* for the strains from 11 sampled Spanish provinces.

Province^a^	N^b^	Na^c^	A^d^	N_H_^e^	H_E_^f^
**Alicante**	22	33	1.30	8	0.059
**Badajoz**	57	62	2.37	24	0.344
**Huesca**	38	70	2.56	30	0.311
**Mallorca**	12	26	1.04	2	0.290
**Teruel**	21	52	2.09	14	0.230
**Valencia**	32	34	1.28	9	0.053
**Zaragoza**	42	68	2.48	27	0.281
**Huelva**	6			3	
**Lleida**	3			3	
**Navarra**	2			2	
**Tarragona**	1			1	
**Unknown**	3			1	
**Total**	239		4.30	119	0.363^g^

^a^ Only provinces with n ≥ 12 were selected to calculate diversity indices (Na, A and H_E_).

^b^ N, number of isolates per province.

^c^ Na, number of alleles.

^d^ A, allelic richness calculated by rarefaction method (n = 12).

^e^ N_H_, number of haplotypes in each province.

^f^ H_E_, Nei’s genetic diversity within provinces.

^g^ H_T_, total Nei’s genetic diversity.

The largest GC (i.e. GC1) contained 48 haplotypes and 111 strains, which represent 40 and 46% of the whole collection, respectively. Strains from nine provinces were found in GC1, including all the strains from Valencia, Mallorca and Tarragona. Only those originating from Alicante and Huelva were not linked to this group. Other GCs were much smaller (≤ 16 haplotypes), and six of them were formed by a single haplotype (GC13, GC14, GC15, GC16, GC17 and GC18). Strains from the province of Badajoz, where the disease was first reported in Spain, and Zaragoza split in six and five different GCs, respectively (Fig 2). All but three GCs (GC1, GC2 and GC7) were composed of a small number of strains originating from a single province with five of them containing strains originating from Badajoz. For example, all strains isolated in Alicante were included in GC4. This was correlated with the allelic richness (A) calculated for each province (Table 2), where Alicante, Mallorca and Valencia showed the lowest values (A ≤ 1.3), while Badajoz, Huesca and Zaragoza displayed the highest values (A > 2.3). Also consistent with the delineation of GCs, pairwise comparisons among populations sampled in seven provinces (n ≥ 12) showed highly significant F_ST_ and R_ST_ values (Table 3) supporting a strong genetic differentiation between strains originating from different provinces. Only strains from Mallorca and Zaragoza showed a R_ST_ value slightly significant (*P* < 0.05).

**Table 3:** Genetic differentiation of Spanish strains of *X*. *arboricola* pv. *pruni* from seven provinces (n ≥ 12) estimated by F_ST_ (above the diagonal) and R_ST_ (below the diagonal) pairwise comparisons based on MLVA data.

		**1**	**2**	**3**	**4**	**5**	**6**	**7**
**1**	**Alicante**		0.535***	0.634***	0.907***	0.703***	0.885***	0.643***
**2**	**Badajoz**	0.367***^a^		0.206***	0.379***	0.263***	0,421***	0.238***
**3**	**Huesca**	0.648***	0.231***		0.341***	0.266***	0.345***	0.106***
**4**	**Mallorca**	0.961***	0.341***	0.307***		0.447***	0.573***	0.241***
**5**	**Teruel**	0.821***	0.325***	0.331***	0.458***		0.507***	0.220***
**6**	**Valencia**	0.954***	0.401***	0.448***	0.559***	0.414***		0.176***
**7**	**Zaragoza**	0.679***	0.268***	0.134***	0.091*	0.228***	0.175***	

^a^ Significance level of F_ST_ and R_ST_ pairwise comparisons

*P < 0.05;

**P < 0.01;

***P < 0.001;

^NS^*P* > 0.005 (non-significant).

Haplotypes grouped in 16 CC and 34 singletons (i.e. strains with no SLV). The correspondence between GCs and CCs is highlighted in Fig 2. Single-repeat variants represented 70.7% of the total number of SLVs, suggesting a predominant stepwise mutation model. Double-repeats variants were observed (25.6% of the total SLVs) only with TR05-06, TR33I, TR38II, TR50I and TR58II loci, which are among the most polymorphic ones. A plausible explanation may be that some intermediate allelic states were missing in our strain collection, or perhaps these loci do not strictly follow a strict stepwise mutation model but a two-phase model [26]. Only three triple-repeat variants were found in TR05-06 locus. Variations involving more than three repeats, which may be attributed to recombination events, were not observed in our dataset.

The largest clonal complex (GC1-A) was formed by 23 haplotypes and 70 strains isolated from six different provinces (Huesca, Lleida, Mallorca, Teruel, Valencia and Zaragoza) between 2003 and 2013 (Fig 2). Two other clonal complexes, GC2-A and GC2-B, were constituted by strains from different provinces (Huesca and Zaragoza). There were provinces with all their strains included in a single clonal complex, e.g. all the strains from Mallorca and Valencia that grouped in GC1-A. Most clonal complexes (13 out of 16) grouped only strains from the same province or the same location.

The primary founder in GC1-A (haplotype 29), the haplotype with the highest number of SLVs (n = 10) corresponded to the most frequent haplotype (n = 18), with strains isolated from 4 different provinces (Huesca n = 1, Lleida n = 1, Valencia n = 12 and Zaragoza n = 4) (Fig 2). In addition, two other haplotypes sharing strains isolated from different provinces were identified in this CC: haplotype 30 composed by eight strains isolated from Mallorca (n = 2) and Valencia (n = 6) and haplotype 32 formed by three strains from Huesca (n = 1) and Zaragoza (n = 2).

Sixty-three strains isolated in seven Spanish nurseries located in five different provinces were distributed in five different GCs (S1 Table). Among them, 32 strains isolated from nursery plants in Valencia in 2003 and seven strains isolated from nursery plants in Zaragoza between 2004 and 2008 grouped into the major clonal complex (GC1-A). Among those, 13 strains (Valencia n = 12, Zaragoza n = 1) were assigned to the primary founder haplotype, suggesting a probable transmission of contaminated plant material by human activities. Fourteen strains were isolated in a single nursery located in Badajoz in 2002 showing very different MLVA profiles. Indeed, two of them clustered in GC1 and the other ones in GC6, suggesting a possible polyclonal or multiple introduction(s) through the same nursery. Other strains from nursery plants were clearly separated with different allelic profiles, such as strains from Zaragoza clustering in GC8 or strains from Huesca and Lleida nurseries in GC13 and GC16, respectively.

Six haplotypes grouped strains isolated both in nurseries and orchards. For three of them the corresponding nurseries and the orchards were located in the same province (two in Badajoz and the other one in Zaragoza). For the three other cases, the nurseries were located in different provinces from those of the orchards.

### MLVA genotyping on a worldwide collection

**Fig 3:**
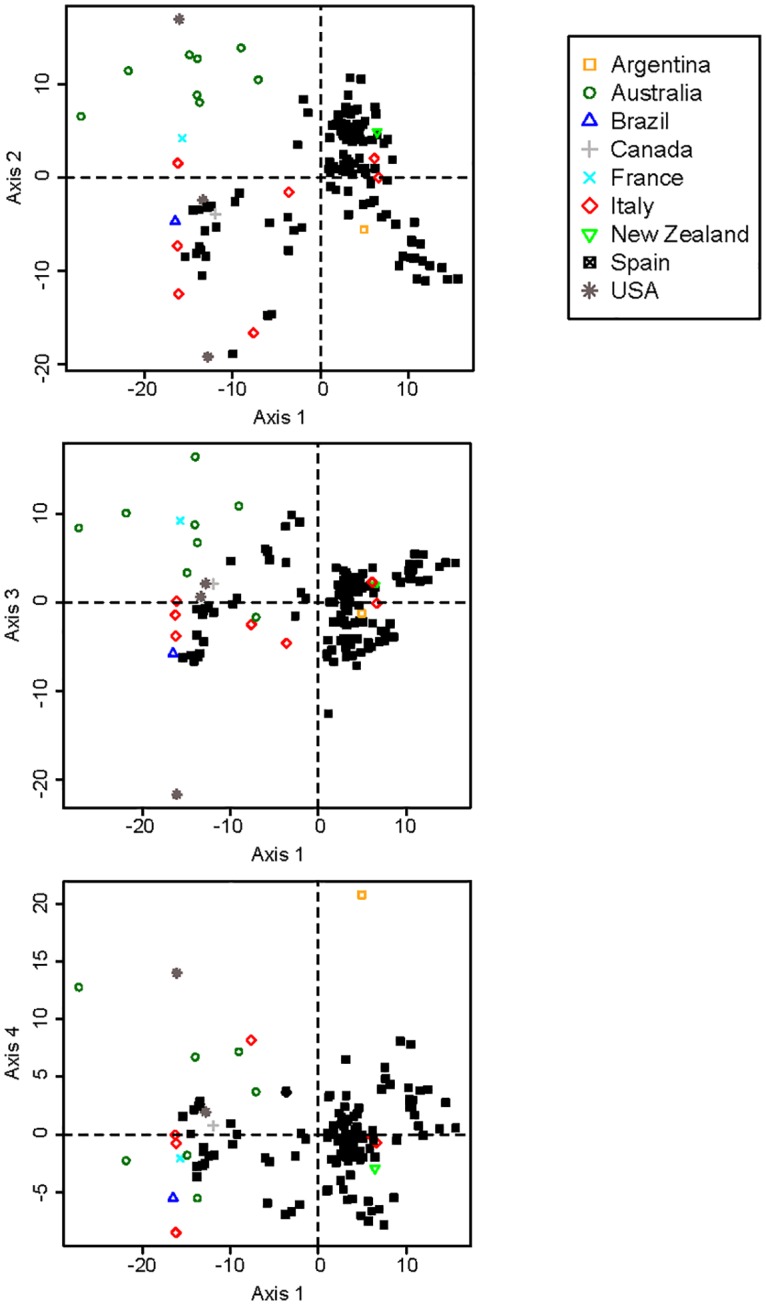
MDS representation of the distances among 264 strains of *X*. *arboricola* pv. *pruni*. Countries of origin are represented by different symbols. MDS axes 1–2, 1–3 and 1–4 described 59.3%, 47.4% and 44.4% of the total variation, respectively.

The 25 *X*. *arboricola* pv. *pruni* strains from our world collection were classified in 23 haplotypes, which means that almost every strain formed a unique haplotype. No haplotype was shared by strains from different countries. MLVA profiles clearly separated the 239 Spanish strains from the world strain collection, showing in most of the cases differences in a range of 4 to 15 loci, even within strains originated from a same country (haplotypes from Australia resulted from 5 to 9-loci variants) (Fig 3). Only two strains, CFBP 3894 from New Zealand and CFBP 5530 from Italy, were SLV (TR79I) and double locus variants (DLV) (TR33I and TR67II) of a Spanish haplotype, respectively, as shown by their close genetic distance on the MDS (Fig 3).

## Discussion

The understanding of the invasion routes, biology and epidemiology of plant pathogens is of striking importance in order to elucidate which are the main factors involved in the success of invasions and to develop strategies aiming at preventing new introductions. Traditionally, two types of methods are employed to retrace invasion routes: direct methods, based on recorded data by quarantine services or agricultural agencies and indirect methods, based on genetic data obtained by the use of molecular markers [49]. In this study we have tried combine both of them, using all the available information from the 239 Spanish strains analyzed.

### MLVA is sufficiently resolutive for molecular epidemiology analyses of *X*. *arboricola* pv. *pruni* over small spatial or temporal scales

The analysis of literature data suggested that *X*. *arboricola* pv. *pruni* is a monomorphic bacterium and all available genotyping techniques but MLVA are not suitable for deciphering the genetic structure of strains during outbreaks. MLVA had been used to distinguish among *X*. *arboricola* pathovars [33] and for assessing the genetic diversity among Dutch strains of *X*. *arboricola* pv. *pruni* isolated from of *P*. *lauroceraus* [35]. Using a comprehensive strain collection, we implemented and evaluated a MLVA scheme derived and improved from Cesbron et al. [33] in order to assess the genetic structure of *X*. *arboricola* pv. *pruni*, eleven years after its emergence in Spain [8].

Around 50% of the Spanish strains were discriminated as different haplotypes, which confirms the appropriateness of MLVA as a genotyping tool for small-scale analyses of this monomorphic pathogen in a relatively small geographical area compared to other techniques such as MLST [17]. A set of 11 Spanish strains shown genetically diverse based on MLVA (i.e. assigned to different GCs) constituted a single MLST haplotype using four housekeeping genes (Garita-Cambronero et al., personal communication).

Conversely and consistent with earlier data on *X*. *citri* pv. *citri* [29, 50] and *X*. *oryzae* [31], our MLVA scheme, which is mainly based on microsatellites, was not appropriate for understanding deep phylogenies and therefore strain relatedness at a global scale.

Our study did not aim at analyzing the role of the hosts in structuring the genetic diversity of *X*. *arboricola* pv. *pruni* because the strain collection available could not allow to extensively address this question. However, based on partial data that have been analyzed for this purpose, no strong or clear structure by the host was observed (data not shown) and several hosts from different provinces shared populations that were not statistically differentiated.

### MLVA supports multiple introductions of *X*. *arboricola* pv. *pruni* in Spain

Since the original description of bacterial spot disease of stone fruits and almond in Badajoz in 2002, the outbreaks caused by *X*. *arboricola* pv. *pruni* in Spain are likely the result of multiple and/or polyclonal introductions. A scenario involving multiple introductions of a pest is a common phenomenon in invasions [51], and it has already been described in some plant pathogenic bacteria such as *Erwinia amylovora* [52, 53].

This hypothesis was suggested by several independent pieces of evidence. Firstly, the 239 Spanish strains were assigned to 18 conservatively delineated GCs (i.e. strains in a network linked by up to quadruple-locus variations were assigned to a single GC). Secondly, pairwise comparisons among populations from Spanish provinces showed highly significant values of F_ST_ and R_ST_ among seven provinces, indicating a strong genetic differentiation. Thirdly, the assignation of isolates collected in 2002 in the Badajoz province further suggested that the initial establishment of the pathogen in Spain was caused by at least four distinct GCs (GC1, GC6, GC9 and GC12).

Few studies focus on the genetic diversity of local emerging strains of a *Xanthomonas* species within the first years following its detection. The situation observed in Spain for *X*. *arboricola* pv. *pruni* was strikingly different from the one reported for *X*. *citri* pv. *citri* strains having emerged in Mali and Burkina Faso [50]. In the latter study, strains from two genetic lineages were identified but only one was detected in commercial citrus nurseries making it markedly prevalent likely through the massive spread of diseased propagative plant material. As a result, a large majority of emerging strains clustered as a single large CC and all epidemic strains would have been assigned to a single GC using the same criteria for GC delineation as herein. On the contrary, the genetic structure revealed in the present study was markedly different, suggestive of more or less concomitant outbreaks having a distinct origin.

Some GCs (i.e. GC3, GC4 and GC10) were formed by a few strains that were isolated in the same orchard and in the same year. These groups of strains showed MLVA profiles completely different to the rest of Spanish strains suggesting that these outbreaks were spatially restricted. These groups are probably the result of local dispersal as a result of natural dispersion by rain and wind or mechanical transmission during agricultural practices within the orchard. Interestingly, strains from these GCs have not been detected from nurseries.

### MLVA together with records from plant protection agencies support the role of nurseries in the introduction and spread of *X*. *arboricola* pv. *pruni* in Spain

Nursery strains analyzed herein clustered in five GCs. Several strains from nurseries in different provinces grouped in the major CC (found in cluster GC1). Interestingly, the primary founder in this CC shared strains from different provinces that were isolated both from two nurseries and several orchards. Field investigations indicated that there had been an exchange of plant material between these two nurseries located in Valencia and Zaragoza, from which strains isolated in 2003 and 2004 respectively, were assigned to this primary founder (haplotype 29). Consistent with these results, several interceptions of contaminated plant material have been reported in Spanish nurseries and most of them consisted of symptomless plants with latent infections [5]. *X*. *arboricola* pv. *pruni* has been detected in seedlings of nectarine, Japanese plum and different stone fruit rootstocks, some of them originating from Italy where the disease is endemic in some regions [9]. A striking example of an interception was documented in GC8, which is composed of four strains from the rootstocks Garnem and Cadaman^®^. All of them were isolated in a nursery from Zaragoza in 2010 but the plants were produced in an Italian nursery (unpublished data). Such strains presented an allelic profile very different from the rest of the Spanish strains.

The largest CC identified herein was formed by strains originated from six different provinces. Some of them, as Huesca, Lleida, Teruel and Zaragoza belong to one of the greatest stone fruit producing areas in Spain, and some of the orchards where the strains were collected are separated by distances ranging from 5 to 30 km. Although natural spread of *X*. *arboricola* pv. *pruni* may occur within orchards during rain showers and hail storms [9, 54, 55], its spread has not been documented over such large distances. To our knowledge, the sole documented case involving extreme weather events (not reported in Spain) was described for *X*. *citri* pv. *citri* in a subtropical environment by Irey et al. [56]. Contaminated nursery plant material may have been associated with such long distance dissemination.

*X*. *arboricola* pv. *pruni* is a pathogen which can survive epiphytically or endophytically in different organs of the plants, such as leaves, twigs, buds, flowers and fruits at low concentration, without causing symptoms of bacterial spot disease [14, 57, 58]. These populations are a potential inoculum source for the development of the disease when weather conditions are favorable and may be transported between nurseries or from them to orchards inadvertently. Our results suggest that nursery plant material has contributed to spread *X*. *arboricola* pv. *pruni* in Spain and that the current inspection procedure consisting of visual observations of typical symptoms in plants in nurseries is insufficient to ascertain their sanitary status. Consequently, survey and sampling protocols combined with high accuracy detection techniques should be applied [59, 60]. There is a need to produce new research data combining microbial ecology and genotyping to improve the knowledge on the biological significance of *X*. *arboricola* pv. *pruni* epiphytic and endophytic populations as a primary inoculum source. Assessing mutation rates of TR markers in future experimental evolution studies would also be valuable.

## Supporting Information

S1 Fig Nucleotide sequence in *Xanthomonas arboricola* pv. *pruni* strain CFBP 3894 of TR loci that were excluded from the analysis or for which new primer combinations were designed. (PDF)

S1 Table *Xanthomonas arboricola* pv. *pruni* strains isolated from 11 provinces in Spain. (PDF)

S2 Table *X*. *arboricola* pv. *pruni* strains from international collections. (PDF)
