# Supplementary material for: Multilocus Variable Number of Tandem Repeat Analysis Reveals Multiple Introductions in Spain of Xanthomonas arboricola pv. pruni, the Causal Agent of Bacterial Spot Disease of Stone Fruits and Almond
Source: PLoS One. 2016 Sep 26;11(9):e0163729. doi: 10.1371/journal.pone.0163729 (PMC5036818; doi:10.1371/journal.pone.0163729)
Supplement: S1 Fig — (PDF) [file pone.0163729.s001.pdf]

TR19I

GATTGACGGCACCCACACAG CGTGCCCACCTTT CGATTCC CGATTCC CGATTCC CGATTCC
CCAATCC TCAATCC TCAATCC TCAATCC TCAATCC TCAATCC TCAATCC
CCACCACGCATGC ACCACGGCACAACGTCCTGG

TR54I

CTGCATCGGCCTGATCGTGG CCGGGGTGATCGGGCTGAAGCTGGTGGGGTAGT
CGTGGTCAGCCCTG CGTGATCAGCCCTG CGTGGTCAGCCC
CACTGCGTGGGCCTGCCTCCCAAGATTTGCTATGCAAACCTTGGGCCCCACTTCGTAGCAGC
CTATCCCCCCGCCTTCGGCGCGCCCCCTTAACAGAAGGGGGCTCTGCTCCAG
TGGGGTGTTGCGAGGTGTGGGGCATAGTTTGCTAGGCGCCGCCA GCCGCCA GCCGCCA
GCCGCCAATCC CCAATCC CCAATCC CCAATCC CCAATCC CCAATCC CCA CCGGCT
ACAGCCGGTACGCCACCGCTTT

TR05II/TR06II

*GTGCAGCACCAGCCAAAGGCAGTTGCATCAGACCCCAGCGCCG****CAGATGCTGTCCCGAT***
TCCCGAT TCCCGAT TCCCGAT TCCCGAT TCCCGAT TCCCGAT TCCCGAT TCCCGAT
TCCCAAT CCCCGAT TCCCAAT CCCCAAT CCCCAAT CCCCAAT CCCCAAT CCCCAAT
CCCCAAT CCCCA GCCTATGA**CCTTCCGCGAACCCGTCGAC**

TR67II

AGCTCGCAACTGCTTTTCCCGA TTCCCGA TTCCCGA TTCCCGA TTCCCGA TTCCCGA
TTCCCGA TTCCCGA TTCCCGA TTCCCGA ATCCCGA TTCCCGA TTCCC
AGCCCCCAATATGCGAAACTTCCACGATGACAATCGTTCTCATCGCGTTCGCCTTGTATC

TR18II

CCGGCATTTTTTTGGAATCG GGAATCG GGAATCG GGAATCG GGAATCG GGAATCG
GGAATC GGGAATCG GGAATCG GAGAGGCG GGAATCG GAGAGGCG
GGAATCGCAGAAGCGGGGTGTCGCTCTTGCTCTACCTATTCCCCATTCCCAATTCCCGATTCC
CAGCCCTCAAAGCATCCGGCTGATCGTGAA

FIG S1 Nucleotide sequence in *Xanthomonas arboricola* pv. pruni strain CFBP 3894 of TR loci developed by Cesbron et al. [33] that were excluded from the analysis (TR19I and TR54I) or for which new primer combinations were designed (other loci).

Color blocks indicate TRs differing in nucleotide sequence. Underlined regions correspond to primers designed by Cesbron et al. [33]. For TR05II and TR06II, the bold and italicized regions correspond to TR05II and TR06II, respectively. Plain and dashed underlined regions correspond to primers designed by Cesbron et al. [33] for TR05II and TR06II, respectively.
