# Supplementary material for: Multilocus Variable Number of Tandem Repeat Analysis Reveals Multiple Introductions in Spain of Xanthomonas arboricola pv. pruni, the Causal Agent of Bacterial Spot Disease of Stone Fruits and Almond
Source: PLoS One. 2016 Sep 26;11(9):e0163729. doi: 10.1371/journal.pone.0163729 (PMC5036818; doi:10.1371/journal.pone.0163729)
Supplement: S1 Table — (PDF) [file pone.0163729.s002.pdf]

S1 Table. *Xanthomonas arboricola* pv. *pruni* strains isolated from 11 provinces in Spain.

Strain[a]	Province	Host of isolation	Cultivar	Year of isolation	Origin	Haplotype number	Genetic cluster (GC)
IVIA 3161.2-1[b]	Alicante	Almond	Rumbeta	2006	Orchard	5	4
IVIA 3161.2-2	Alicante	Almond	Rumbeta	2006	Orchard	5	4
IVIA 3161.4	Alicante	Almond	Rumbeta	2006	Orchard	5	4
IVIA 3162.1[c]	Alicante	Almond	Rumbeta	2006	Orchard	46	4
IVIA 3162.2	Alicante	Almond	Rumbeta	2006	Orchard	7	4
IVIA 3162.3	Alicante	Almond	Rumbeta	2006	Orchard	7	4
IVIA 3162.4-5	Alicante	Almond	Rumbeta	2006	Orchard	5	4
IVIA 3162.4-6	Alicante	Almond	Rumbeta	2006	Orchard	7	4
IVIA 3177.1-1-1	Alicante	Almond	Rumbeta	2006	Orchard	5	4
IVIA 3177.1-6-2	Alicante	Almond	Rumbeta	2006	Orchard	5	4
IVIA 3177.3-4-3	Alicante	Almond	Rumbeta	2006	Orchard	40	4
IVIA 3177.3-8-4	Alicante	Almond	Rumbeta	2006	Orchard	48	4
IVIA 3177.3-10-5	Alicante	Almond	Rumbeta	2006	Orchard	5	4
IVIA 3181.3-1-6	Alicante	Almond	Rumbeta	2006	Orchard	47	4
IVIA 3181.3-1-7	Alicante	Almond	Rumbeta	2006	Orchard	6	4

IVIA 3181.3-1-8	Alicante	Almond	Rumbeta	2006	Orchard	6	4
IVIA 3181.3-3-1	Alicante	Almond	Rumbeta	2006	Orchard	4	4
IVIA 3181.3-3-2	Alicante	Almond	Rumbeta	2006	Orchard	4	4
IVIA 3181.3-3-3	Alicante	Almond	Rumbeta	2006	Orchard	4	4
IVIA 3181.3-3-4	Alicante	Almond	Rumbeta	2006	Orchard	4	4
IVIA 3181.3-3-5	Alicante	Almond	Rumbeta	2006	Orchard	4	4
IVIA 3181.3-3-6	Alicante	Almond	Rumbeta	2006	Orchard	4	4
IVIA 2626.1[c]	Badajoz	Japanese plum	Fortune	2002	Orchard	2	6
IVIA 2626.2	Badajoz	Japanese plum	Fortune	2002	Orchard	2	6
IVIA 2626.3	Badajoz	Japanese plum	Fortune	2002	Orchard	13	1
IVIA 2626.4	Badajoz	Japanese plum	Fortune	2002	Orchard	64	1
IVIA 2626.5	Badajoz	Japanese plum	Fortune	2002	Orchard	2	6
IVIA 2626.6[b]	Badajoz	Japanese plum	Fortune	2002	Orchard	14	1
IVIA 2626.7	Badajoz	Japanese plum	Fortune	2002	Orchard	13	1
IVIA 2626.8a	Badajoz	Japanese plum	Fortune	2002	Orchard	43	6
IVIA 2626.8b	Badajoz	Japanese plum	Fortune	2002	Orchard	50	6
IVIA 2626.10	Badajoz	Japanese plum	Fortune	2002	Orchard	14	1

IVIA 2647.1-2	Badajoz	Japanese plum	Larry Ann	2002	Nursery	44	6
IVIA 2647.1-3	Badajoz	Japanese plum	Larry Ann	2002	Nursery	2	6
IVIA 2647.1-6	Badajoz	Japanese plum	Larry Ann	2002	Nursery	2	6
IVIA 2647.1-7	Badajoz	Japanese plum	Larry Ann	2002	Nursery	2	6
IVIA 2647.1-8	Badajoz	Japanese plum	Larry Ann	2002	Nursery	1	6
IVIA 2647.1-9	Badajoz	Japanese plum	Larry Ann	2002	Nursery	1	6
IVIA 2647.1-10	Badajoz	Japanese plum	Larry Ann	2002	Nursery	2	6
IVIA 2647.1-12a	Badajoz	Japanese plum	Larry Ann	2002	Nursery	2	6
IVIA 2647.1-12b	Badajoz	Japanese plum	Larry Ann	2002	Nursery	2	6
IVIA 2647.1-13	Badajoz	Japanese plum	Larry Ann	2002	Nursery	2	6
IVIA 2647.1-14	Badajoz	Japanese plum	Larry Ann	2002	Nursery	2	6
IVIA 2647.1-15	Badajoz	Japanese plum	Larry Ann	2002	Nursery	2	6
IVIA 2647.3-1	Badajoz	Japanese plum	Friar	2002	Nursery	13	1
IVIA 2647.3-2	Badajoz	Japanese plum	Friar	2002	Nursery	13	1
IVIA 2649.1	Badajoz	Japanese plum	Friar	2002	Orchard	16	9
IVIA 2649.2	Badajoz	Japanese plum	Friar	2002	Orchard	66	9
IVIA 2649.3	Badajoz	Japanese plum	Friar	2002	Orchard	16	9

IV IA 2649.4	Badajoz	Japanese plum	Friar	2002	Orchard	16	9
IVIA 2649.7	Badajoz	Japanese plum	Friar	2002	Orchard	68	9
IVIA 2649.10	Badajoz	Japanese plum	Friar	2002	Orchard	16	9
IVIA 2667	Badajoz	Japanese plum	Friar	2002	Orchard	3	12
IVIA 2758.1	Badajoz	Japanese plum		2003	Orchard	9	10
IVIA 2758.2	Badajoz	Japanese plum		2003	Orchard	52	10
IVIA 2758.3	Badajoz	Japanese plum		2003	Orchard	9	10
IVIA 2795	Badajoz	Japanese plum	Larry Ann	2003	Orchard	53	10
IVIA 3355.1	Badajoz	Japanese plum	Larry Ann	2007	Orchard	3	12
IVIA 3355.2	Badajoz	Japanese plum	Larry Ann	2007	Orchard	45	12
IVIA 3373.1a	Badajoz	Japanese plum	Friar	2007	Orchard	18	9
IVIA 3373.1b	Badajoz	Japanese plum	Friar	2007	Orchard	18	9
IVIA 3373.2a	Badajoz	Japanese plum	Friar	2007	Orchard	18	9
IVIA 3373.2b	Badajoz	Japanese plum	Friar	2007	Orchard	18	9
IVIA 3374a	Badajoz	Japanese plum	Angeleno	2007	Orchard	18	9
IVIA 3374b	Badajoz	Japanese plum	Angeleno	2007	Orchard	18	9
IVIA 3378.2-2	Badajoz	Nectarine	Zeeglo	2007	Orchard	18	9

IVIA 3378.3	Badajoz	Nectarine	Zeeglo	2007	Orchard	18	9
IVIA 3437.5	Badajoz	Japanese plum		2008	Orchard	11	5
IVIA 3437.6	Badajoz	Japanese plum		2008	Orchard	10	5
IVIA 3437.7	Badajoz	Japanese plum		2008	Orchard	10	5
IVIA 3438.8	Badajoz	Japanese plum		2008	Orchard	11	5
IVIA 3438.10	Badajoz	Japanese plum		2008	Orchard	11	5
IVIA 3439.1	Badajoz	Japanese plum		2008	Orchard	11	5
IVIA 3439.3	Badajoz	Japanese plum		2008	Orchard	56	5
IVIA 3439.5	Badajoz	Japanese plum		2008	Orchard	11	5
IVIA 3439.7	Badajoz	Japanese plum		2008	Orchard	57	5
IVIA 3439.8	Badajoz	Japanese plum		2008	Orchard	54	5
IVIA 3741	Badajoz	Japanese plum	Larry Ann	2010	Orchard	62	5
IVIA 3742	Badajoz	Japanese plum	Golden Plum	2010	Orchard	61	5
IVIA 4266.1	Huelva	Japanese plum	Black Late	2012	Orchard	35	11
IVIA 4266.2	Huelva	Japanese plum	Black Late	2012	Orchard	35	11
IVIA 4266.7	Huelva	Japanese plum	Black Late	2012	Orchard	34	11
IVIA 4266.9	Huelva	Japanese plum	Black Late	2012	Orchard	34	11

IVIA 4286.1-1	Huelva	Japanese plum	Black Late	2012	Orchard	49	14
IVIA 4286.2-2	Huelva	Japanese plum	Black Late	2012	Orchard	34	11
IVIA 3487.1	Huesca	Apricot		2008	Orchard	72	3
IVIA 3487.3	Huesca	Apricot		2008	Orchard	22	3
IVIA 3487.4	Huesca	Apricot		2008	Orchard	21	3
IVIA 3489.2	Huesca	Japanese plum		2008	Orchard	71	3
IVIA 3489.4	Huesca	Japanese plum		2008	Orchard	21	3
IVIA 3490.1	Huesca	Japanese plum		2008	Orchard	20	3
IVIA 3490.2	Huesca	Japanese plum		2008	Orchard	20	3
IVIA 3490.3	Huesca	Japanese plum		2008	Orchard	21	3
IVIA 3490.4	Huesca	Japanese plum		2008	Orchard	73	3
IVIA 3491.7	Huesca	Japanese plum		2008	Orchard	70	3
IVIA 3491.8	Huesca	Japanese plum		2008	Orchard	69	3
IVIA 3492.2	Huesca	Japanese plum		2008	Orchard	19	3
IVIA 3492.3	Huesca	Japanese plum		2008	Orchard	19	3
IVIA 3604.2	Huesca	Almond		2009	Orchard	114	2
IVIA 3604.7	Huesca	Almond		2009	Orchard	12	1

IVIA 3604.10	Huesca	Almond		2009	Orchard	107	2
IVIA 3604.12	Huesca	Almond		2009	Orchard	108	2
IVIA 4165.15	Huesca	Almond	Guara	2011	Orchard	32	1
IVIA 4165.21	Huesca	Almond	Guara	2011	Orchard	113	2
IVIA 4166.12	Huesca	Almond	Guara	2011	Orchard	29	1
IVIA 4166.28	Huesca	Almond	Guara	2011	Orchard	92	1
IVIA 4491.1	Huesca	Almond		2013	Orchard	116	2
IVIA 4491.2	Huesca	Almond		2013	Orchard	117	2
IVIA 4491.3	Huesca	Almond		2013	Orchard	110	2
IVIA 4492.1	Huesca	Almond		2013	Orchard	111	2
IVIA 4492.2	Huesca	Almond		2013	Orchard	112	2
IVIA 4492.3	Huesca	Almond		2013	Orchard	118	2
IVIA 4492.4	Huesca	Almond		2013	Orchard	119	2
IVIA 4493	Huesca	Almond		2013	Orchard	25	1
CITA 21	Huesca	Almond		2008	Orchard	22	3
CITA 38	Huesca	Almond		2009	Orchard	12	1
CITA 56	Huesca	Almond		2010	Orchard	12	1

CITA 63	Huesca	Almond	Guara	2010	Orchard	115	2
CITA 116	Huesca	Almond		2011	Orchard	105	2
CITA 145	Huesca	Almond		2012	Nursery	41	13
CITA 148	Huesca	Almond	Guara	2012	Orchard	82	1
CITA 170	Huesca	Almond	Vayro	2013	Orchard	51	18
CITA 173	Huesca	Almond	Vayro	2013	Orchard	25	1
IVIA 3479.1	Lleida	Peach	Ryan Sun	2008	Orchard	81	1
CITA 91	Lleida	Peach		2011	Nursery	88	16
CITA 143	Lleida	Almond		2012	Orchard	29	1
IVIA 3704.2[c]	Mallorca	Japanese plum	Larry Ann	2010	Orchard	31	1
IVIA 3704.3	Mallorca	Japanese plum	Larry Ann	2010	Orchard	31	1
IVIA 3704.5	Mallorca	Japanese plum	Larry Ann	2010	Orchard	31	1
IVIA 3704.6	Mallorca	Japanese plum	Larry Ann	2010	Orchard	31	1
IVIA 3704.7	Mallorca	Japanese plum	Larry Ann	2010	Orchard	31	1
IVIA 3704.8	Mallorca	Japanese plum	Larry Ann	2010	Orchard	31	1
IVIA 3704.9	Mallorca	Japanese plum	Larry Ann	2010	Orchard	30	1
IVIA 3704.10	Mallorca	Japanese plum	Larry Ann	2010	Orchard	31	1

IVIA 3704.11	Mallorca	Japanese plum	Larry Ann	2010	Orchard	30	1
IVIA 3704.12	Mallorca	Japanese plum	Larry Ann	2010	Orchard	31	1
IVIA 3705.6	Mallorca	Japanese plum	Fortune	2010	Orchard	31	1
IVIA 3705.7	Mallorca	Japanese plum	Fortune	2010	Orchard	31	1
CITA 53	Navarra	Almond	Guara	2009	Orchard	100	1
CITA 77	Navarra	Almond	Guara	2010	Orchard	59	7
IVIA 4113[c]	Tarragona	Almond	Guara	2011	Orchard	78	1
IVIA 4330[c]	Teruel	Almond	Guara	2009	Orchard	93	1
IVIA 4490.1	Teruel	Almond		2013	Orchard	15	1
IVIA 4490.2	Teruel	Almond		2013	Orchard	15	1
IVIA 4494.1	Teruel	Apricot		2013	Orchard	36	1
IVIA 4494.2	Teruel	Apricot		2013	Orchard	36	1
IVIA 4523.1	Teruel	Peach	Royal Lee	2013	Orchard	65	1
IVIA 4523.2	Teruel	Peach	Royal Lee	2013	Orchard	36	1
IVIA 4523.3	Teruel	Peach	Royal Lee	2013	Orchard	102	1
IVIA 4524.1	Teruel	Peach	Royal Lee	2013	Orchard	96	1
IVIA 4524.2	Teruel	Peach	Royal Lee	2013	Orchard	87	1

IVIA 4524.3	Teruel	Peach	Royal Lee	2013	Orchard	37	1
IVIA 4524.4	Teruel	Peach	Royal Lee	2013	Orchard	85	1
IVIA 4524.5	Teruel	Peach	Royal Lee	2013	Orchard	36	1
CITA 34	Teruel	Almond	Guara	2009	Orchard	26	1
CITA 35	Teruel	Almond	Guara	2009	Orchard	58	7
CITA 36	Teruel	Almond	Guara	2009	Orchard	26	1
CITA 68	Teruel	Almond	Guara	2010	Orchard	99	1
CITA 138	Teruel	Almond		2012	Orchard	37	1
CITA 172	Teruel	Almond	Guara	2013	Orchard	15	1
CITA 174	Teruel	Flat peach		2013	Orchard	97	1
CITA 175	Teruel	Flat peach		2013	Orchard	98	1
IVIA 2826.1	Valencia	Japanese plum	Anna Gold	2003	Nursery	29	1
IVIA 2826.2[b]	Valencia	Japanese plum	Anna Gold	2003	Nursery	28	1
IVIA 2826.3	Valencia	Japanese plum	Anna Gold	2003	Nursery	28	1
IVIA 2826.4	Valencia	Japanese plum	Anna Gold	2003	Nursery	30	1
IVIA 2826.5	Valencia	Japanese plum	Anna Gold	2003	Nursery	29	1
IVIA 2826.6	Valencia	Japanese plum	Anna Gold	2003	Nursery	30	1

IVIA 2826.7	Valencia	Japanese plum	Anna Gold	2003	Nursery	30	1
IVIA 2826.8	Valencia	Peach	Zephir	2003	Nursery	79	1
IVIA 2826.9	Valencia	Peach	Zephir	2003	Nursery	86	1
IVIA 2826.10	Valencia	Peach	Zephir	2003	Nursery	30	1
IVIA 2826.11	Valencia	Peach	Zephir	2003	Nursery	30	1
IVIA 2832.4b	Valencia	Peach	Plamaguel	2003	Nursery	29	1
IVIA 2832.5	Valencia	Peach	Plamaguel	2003	Nursery	29	1
IVIA 2832.7	Valencia	Japanese plum	Angeleno	2003	Nursery	27	1
IVIA 2832.10	Valencia	Japanese plum	Angeleno	2003	Nursery	27	1
IVIA 2832.17	Valencia	Japanese plum	Larry Ann	2003	Nursery	28	1
IVIA 2832.19	Valencia	Japanese plum	Larry Ann	2003	Nursery	28	1
IVIA 2832.20	Valencia	Peach	Zephir	2003	Nursery	80	1
IVIA 2832.21	Valencia	Peach	Zephir	2003	Nursery	28	1
IVIA 2832.22	Valencia	Japanese plum	Anna Gold	2003	Nursery	29	1
IVIA 2832.24	Valencia	Japanese plum	Anna Gold	2003	Nursery	29	1
IVIA 2832.25	Valencia	Peach	58CC70	2003	Nursery	29	1
IVIA 2832.26	Valencia	Peach	58CC70	2003	Nursery	29	1

IVIA 2832.27	Valencia	Peach	58CC70	2003	Nursery	29	1
IVIA 2832.30a	Valencia	Peach x Chinese peach	Cadaman®	2003	Nursery	29	1
IVIA 2832.30b	Valencia	Peach x Chinese peach	Cadaman®	2003	Nursery	29	1
IVIA 2835.4[c]	Valencia	Peach	Zephir	2003	Nursery	28	1
IVIA 2835.5	Valencia	Peach	Zephir	2003	Nursery	28	1
IVIA 2835.6	Valencia	Peach	Zephir	2003	Nursery	90	1
IVIA 2835.7	Valencia	Japanese plum	Anna Gold	2003	Nursery	29	1
IVIA 2835.8	Valencia	Japanese plum	Anna Gold	2003	Nursery	83	1
IVIA 2835.9	Valencia	Japanese plum	Anna Gold	2003	Nursery	30	1
IVIA 3467.1	Zaragoza	Peach		2008	Orchard	109	2
IVIA 3467.2	Zaragoza	Peach		2008	Orchard	67	1
IVIA 3724.3	Zaragoza	Peach x Chinese peach	Barrier	2009	Nursery	103	8
IVIA 3767.1[d]	Zaragoza	Almond x peach	Garnem	2010	Nursery	104	8
IVIA 3767.2[d]	Zaragoza	Almond x peach	Garnem	2010	Nursery	101	8
IVIA 3767.3[d]	Zaragoza	Peach x Chinese peach	Cadaman®	2010	Nursery	38	8
IVIA 3767.4[cd]	Zaragoza	Peach x Chinese peach	Cadaman®	2010	Nursery	38	8
CITA 4	Zaragoza	Peach	Catherine	2004	Nursery	17	1

CITA 5	Zaragoza	Peach	Catherine	2004	Nursery	17	1
CITA 6	Zaragoza	Peach	Catherine	2004	Nursery	91	1
CITA 7	Zaragoza	Peach		2004	Nursery	95	1
CITA 8	Zaragoza	Peach		2004	Nursery	29	1
CITA 12	Zaragoza	Peach	Honey Royal	2008	Nursery	32	1
CITA 13	Zaragoza	Peach	Gardeta	2008	Nursery	84	1
CITA 15	Zaragoza	Peach	Ryan Sun	2008	Nursery	32	1
CITA 19	Zaragoza	Peach		2008	Nursery	23	1
CITA 22	Zaragoza	Peach		2008	Experimental plot	23	1
CITA 23	Zaragoza	Peach	Evaisa	2008	Experimental plot	29	1
CITA 24	Zaragoza	Almond	Ayles	2008	Experimental plot	29	1
CITA 25	Zaragoza	Almond		2008	Experimental plot	23	1
CITA 26	Zaragoza	Almond x peach	Monegro	2008	Experimental plot	39	2
CITA 29	Zaragoza	Peach		2008	Experimental plot	29	1
CITA 37	Zaragoza	Almond	Guara	2009	Orchard	63	7
CITA 39	Zaragoza	Almond	Guara	2009	Orchard	60	7
CITA 40	Zaragoza	Almond	Guara	2009	Orchard	74	1

CITA 43	Zaragoza	Almond	Guara	2009	Orchard	94	1
CITA 47	Zaragoza	Apricot	Cristali	2009	Experimental plot	77	1
CITA 48	Zaragoza	Japanese plum	El Dorado	2009	Experimental plot	89	1
CITA 54	Zaragoza	Almond		2010	Experimental plot	39	2
CITA 59	Zaragoza	Almond		2010	Orchard	39	2
CITA 64	Zaragoza	Almond		2010	Experimental plot	106	2
CITA 69	Zaragoza	Peach		2010	Experimental plot	24	1
CITA 75	Zaragoza	Almond		2010	Experimental plot	33	1
CITA 76	Zaragoza	Almond	Guara	2010	Orchard	42	17
CITA 87	Zaragoza	Almond		2010	Experimental plot	33	1
CITA 89	Zaragoza	Almond	Guara	2010	Orchard	55	7
CITA 114	Zaragoza	Almond		2011	Experimental plot	33	1
CITA 118	Zaragoza	Almond		2011	Nursery	75	1
CITA 121	Zaragoza	Peach x almond		2011	Experimental plot	24	1
CITA 123	Zaragoza	Almond	Guara	2011	Orchard	76	1
CITA 153	Zaragoza	Almond x peach	Garnem	2012	Experimental plot	24	1
CITA 154	Zaragoza	Almond		2012	Orchard	39	2

IVIA 3397.1-1	Unknown	Japanese plum				8	15
IVIA 3397.1-2	Unknown	Japanese plum				8	15
IVIA 3397.2	Unknown	Japanese plum				8	15

[a] IVIA, Instituto Valenciano de Investigaciones Agrarias, Moncada, Valencia, Spain; CITA, Centro de Investigación y Tecnología Agroalimentaria de Aragón, Montañana, Zaragoza, Spain.

[b] Strains deposited in the Collection Française de Bactéries Phytopathogènes (CFBP), INRA, Angers, France.

[c] Strains included in the panel test for preliminary primers screening.

[d] Interception by the Centro de Sanidad y Certificación Vegetal del Gobierno de Aragón (CSCV), Spain, of Garnem and Cadaman® rootstocks plants from an Italian nursery.
