# Supplementary material for: Multilocus Variable Number of Tandem Repeat Analysis Reveals Multiple Introductions in Spain of Xanthomonas arboricola pv. pruni, the Causal Agent of Bacterial Spot Disease of Stone Fruits and Almond
Source: PLoS One. 2016 Sep 26;11(9):e0163729. doi: 10.1371/journal.pone.0163729 (PMC5036818; doi:10.1371/journal.pone.0163729)
Supplement: S2 Table — (PDF) [file pone.0163729.s003.pdf]

S2 Table. ***X. arboricola*** **pv.** ***pruni*** **strains from international collections.**

Strain[a]	Country of origin	Host of isolation	Year of isolation	Haplotype
**CFBP 5229**[b]	Argentina	*Prunus* spp.	1996	W3
**CFBP 5529**[b]	Australia	Peach	1964	W15
**DAR 33337**	Australia	Japanese plum	1978	W18
**DAR 33420**	Australia	Peach	1980	W17
**DAR 41285**	Australia	Peach	1982	W1
**DAR 41286**	Australia	Peach	1982	W1
**DAR 41287**	Australia	Peach	1982	W11
**DAR 56679**	Australia	Apricot	1987	W2
**DAR 56680**	Australia	Japanese plum	1987	W2
**DAR 61729**	Australia	European plum	1988	W16
**DAR 69849**	Australia	Peach x almond	1994	W13
**CFBP 5722**[b]	Brazil	Peach	1991	W9
**CFBP 1311**[b]	Canada			W7
**CFBP 5562**[b]	France	Peach	1995	W14
**CFBP 5530**[b]	Italy	Peach	1989	W21
**ISF 43**	Italy	Japanese plum	1993	W22
**ISF 463**	Italy	Peach	1996	W10
**ISF 464**	Italy	Peach	1996	W6
**ISF 465**	Italy	Peach	1996	W12
**ISF 515**	Italy	Peach	1997	W19
**ISPaVe B4**	Italy	Japanese plum		W4
**CFBP 3894**[bc]	New Zealand	Japanese plum	1953	W20
**CFBP 411**	USA		1963	W8
**CFBP 5720**[b]	USA	Peach	1987	W23
**CFBP 5724**[b]	USA	Almond		W5

[a] CFBP: Collection Française de Bactéries Phytopathogènes, INRA, Angers, France; DAR: Australian Collection of Plant Pathogenic Bacteria, Rydalmere, Australia; ISF:

Culture Collection of C.R.A.: Centro di Ricerca per la Frutticoltura, Roma, Italia; ISPaVe: Istituto di Patologia Vegetale, Bologna, Italia.

[b] Strains included in the panel test.

[c] Pathotype strain.
